# Mechanisms and Prevention Strategies of Macrophage Involvement in the Progression From Hypertension to Heart Failure

**DOI:** 10.1155/ijhy/2618127

**Published:** 2025-06-20

**Authors:** Ningning Zhang, Pengyu Cao, Bojian Wang, Jinting Yang, Lijing Zhao, Wangshu Shao

**Affiliations:** ^1^The Cardiovascular Center, Changzhou No. 2 People's Hospital, The Third Affiliated Hospital of Nanjing Medical University, Changzhou 213164, Jiangsu, China; ^2^Changzhou Medical Center, Nanjing Medical University, Changzhou 213004, Jiangsu, China; ^3^School of Nursing, Jilin University, Changchun 130021, Jilin, China; ^4^The Cardiovascular Center, First Hospital of Jilin University, Changchun 130021, Jilin, China

**Keywords:** exercise training, heart failure, hypertension, macrophages, ventricular remodeling

## Abstract

Hypertensive heart disease, a condition affecting millions worldwide, encompasses a spectrum from uncontrolled hypertension to heart failure. Despite the intricacies of its pathogenic mechanisms, recent attention has been directed toward the role of macrophages in the progression from hypertension to heart failure. Under normal circumstances, macrophages contribute to tissue homeostasis by clearing deceased cells. However, dysregulation during hypertension triggers inflammatory pathways, leading to tissue damage. Oxidative stress and mitochondrial dysfunction are implicated in this process. Exercise training, gaining popularity for its potential in regulating macrophage function, emerges as a promising intervention to improve outcomes in hypertensive heart disease. This review provides a succinct overview of previous research elucidating the involvement of macrophages in the transition from hypertension to heart failure. It underscores the current active areas of investigation and emphasizes the potential of exercise training in mediating macrophage responses, offering a glimpse into a hopeful avenue for therapeutic intervention in this challenging medical condition.

## 1. Introduction

Hypertension, the most prevalent cardiovascular ailment globally, underwent reclassification by the American Heart Association and the American College of Cardiology, setting the threshold at 140/90 mm Hg for persistently elevated systemic arterial blood pressure [1]. The worldwide prevalence of hypertension was steadily rising, projected to reach 60% by 2025 [2]. Uncontrolled hypertension could lead to heart failure (HF), impacting over 64 million individuals globally, with numbers on the rise [3]. Chronic hypertension induced vascular remodeling and blood vessel wall thickening [4], escalating cardiac workload and prompting ventricular hypertrophy, characterized by increased cardiomyocyte volume and alterations in myocardial collagen matrix composition [5]. Ventricular hypertrophy heightened myocardial oxygen consumption, yet coronary artery dilatation fell short, resulting in myocardial ischemia due to increased demand from the thickened myocardium [6]. This cascade of events, including cardiac overload and myocardial ischemia, contributed to cardiac remodeling, fostering HF development.

Macrophages, primitive immune cells ubiquitous in nearly all tissues, underwent classification into two primary categories: tissue-resident macrophages, situated in tissues such as perivascular tissue, heart, and kidney, and monocyte-derived macrophages, infiltrating tissues from circulating blood [7–9]. The majority of resident macrophages established prenatally persisted through local proliferation, with blood monocytes contributing to their replacement. Disruption of homeostasis led not only to the recruitment but also to the permanent replacement of embryonically established resident macrophages by monocyte-derived macrophages, occurring in the myocardium and other organs [10]. Macrophages in the resting state (M0) were generally considered precursors of polarized macrophages and further classified into two main types: classical activated macrophages (M1) and alternative activated macrophages (M2), each exerting distinct immune responses [11].

Hypertensive heart disease represented a spectrum from uncontrolled hypertension to HF. Macrophages played a crucial role in vascular and cardiac healing, maintaining homeostasis for vascular smooth muscle endothelial cells and cardiomyocytes [12–15]. However, macrophage activation was necessary for vascular and cardiac remodeling, influencing the entire progression from hypertension to HF. The specific pathogenic mechanisms underlying this process remained elusive [16–18]. This review aimed to provide a comprehensive overview of the research on immune responses involving macrophages in the spectrum from hypertension to HF, emphasizing the most actively explored areas of interest, as well as investigations into exercise training for modulating macrophages.

## 2. Macrophage Type in Vascular Tissue and Myocardial Tissue

In vascular tissue, three primary macrophage subsets exist [19, 20]: proinflammatory M0 and M1 cells and anti-inflammatory M2 cells. M0 macrophages activate inflammatory vesicles by coexpressing cytokines, such as interleukin-1β (IL-1β) [20]. M1 macrophages express TNF, driving the activation, recruitment, and interactions of immune cells within this phenotype [20]. M2 macrophages, driven by anti-inflammatory pathways such as the STAT6 pathway, primarily express foam cell marker genes such as ABCA1, Abcg1, matrix metalloproteinase (MMP)-9, and TREM-2 [20]. Recent studies have highlighted the significance of TREM-2^(high)^ macrophages [19]. These cells not only regulate lipid metabolism and inhibit vascular inflammation and late calcification [21, 22] but also express molecules that exacerbate plaque rupture [23].

Under pathophysiological conditions, cardiac macrophages have been categorized into two distinctive groups: Ly-6C^(high)^CD206^(−)^CD204^(−)^ classically activated M1 macrophages and Ly-6C^(low)^CD206^(+)^CD204^(+)^ alternatively activated M2 macrophages [24]. Two distinct monocyte-derived cardiac macrophages differently contribute to the healing process against cardiac stress or injury [24]. Ly-6C^(high)^ monocytes are recruited to the injured myocardium through CC chemokine receptor 2 (CCR2) and undergo differentiation into classically activated M1 macrophages [25, 26]. These M1 macrophages are characterized by the production of IL-1β, IL-6, and TNF-α, coupled with phagocytic, proteolytic, and proinflammatory functions. During the early phases of cardiac injury, M1 macrophages engage in phagocytosis and clearance of debris within the heart [11, 24]. In later stages, Ly-6C^(low)^ monocytes are recruited to the myocardium via CX3C chemokine receptor 1 (CX3CR1) and differentiate into M2 macrophages, expressing transforming growth factor-β (TGF-β) and IL-12 [25, 26]. M2 macrophages contribute to an anti-inflammatory response, neovascularization, and myofibroblast activation during the healing process after cardiac injury [11, 24].

## 3. The Role of Macrophages in the Development and Progression From Hypertension to HF

The progression from hypertension to HF is delineated into three distinct stages: hypertension and its associated vasculopathy, hypertensive cardiac hypertrophy, and hypertensive ventricular remodeling. This segment delves into the intricacies of macrophage mechanisms and functions, focusing specifically on their roles within each of these developmental stages.

### 3.1. Hypertension and Its Subsequent Vasculopathy

**Figure 1:**
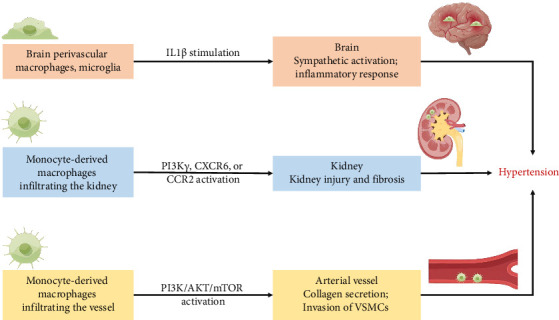
The role of macrophages in the development of hypertension and its subsequent vasculopathy.

The comorbidity-driven systemic proinflammatory state, which triggered microvascular and myocardial dysfunction, emerged as central components of HF pathophysiology [27]. As outlined in Table 1 and Figure 1, both resident macrophages and monocyte-derived macrophages played roles in the development of hypertension and vascular remodeling, albeit through distinct mechanisms of action and function, ultimately contributing to the onset of renal illness and cardiovascular disease.

Firstly, hypertension, a condition intricately linked with systemic inflammation, manifests a critical involvement of the sympathetic nervous system (SNS) in its pathogenesis. In the previous study [28], the burgeoning phase of hypertension in male stroke-prone spontaneously hypertensive rats (SHRSP) revealed an augmentation in both the number of brain perivascular macrophages (PVMs) and circulating levels of IL-1β; the elimination of increased brain PVMs by the intracerebroventricular injection of clodronate liposomes attenuated blood pressure elevation in SHRSP, and the clodronate-treated SHRSP exhibited diminished sympathetic activity accompanied by a reduction in neuronal activity within the sympathetic regulatory nuclei; moreover, extending the scope of the study to Wistar–Kyoto rats (WKY), the administration of clodronate displayed a mitigating effect on the rise in blood pressure and sympathetic nerve activity in response to an acute intravenous injection of IL-1β; these findings demonstrate that brain PVMs contribute to the development of hypertension via sympathetic activation, and brain PVMs may be activated, at least partially, by increased circulating IL-1β [28]. Furthermore, adult microglia, originating from primitive macrophages [58], exhibited a biphasic influence: M2 microglia promoting beneficial repair and M1 microglia causing harmful damage. Resting perivascular microglia, transitioning from a transient proliferative state to a proinflammatory M1 state, contributed to hypertension development [29].

Secondly, macrophage alterations in the kidney played a pivotal role in hypertension pathogenesis. Vitamin D deficiency instigates proinflammatory macrophage infiltration within metabolic tissues, correlating with renin-mediated hypertension. Oh J., et al. tested that impaired vitamin D signaling in macrophages causes hypertension using conditional knockout of the myeloid vitamin D receptor (VDR) in mice (KODMAC) [31]. In the study, the myeloid-specific VDR deficiency promotes vascular and renal macrophage infiltration and increases renin-angiotensin system (RAS) dependent hypertension in mice; the myeloid-specific VDR deficiency induces endoplasmic reticulum stress in KODMAC macrophages, leading to heightened exosome secretion of miR-106b-5p, and in turn, triggers augmented renin production in juxtaglomerular (JG) cells, culminating in hypertension; intriguingly, in wild-type recipient mice transplanted with KODMAC/miR106b^−/−^ bone marrow, the knockout of miR-106b-5p effectively prevents the hypertension and JG cell renin production induced by KODMAC macrophages. These compelling findings conclusively establish that the secretion of macrophage-derived miR-106b-5p, resulting from impaired VDR signaling, substantiates macrophage infiltration within renal vasculature, thereby contributing to inflammation-induced hypertension. Additional research has identified other microRNAs that play significant roles in hypertension and renal pathophysiology. For example, miR-21 has been shown to be upregulated in hypertensive kidneys and contributes to renal fibrosis by targeting genes involved in antifibrotic pathways [59]. Similarly, miR-29 is known to regulate collagen expression, and its downregulation has been associated with increased fibrosis in hypertensive conditions [60]. On the other hand, miR-155 has been implicated in promoting inflammation and renal damage in hypertension by modulating the immune response and macrophage activity [61]. Kidneys, accumulating a large number of macrophages due to hypertension, suffered from macrophage influx via phosphatidylinositol 3 phosphate kinase γ (PI3Kγ) or C-X-C chemokine receptor 6 (CXCR6) activation, resulting in kidney injury and fibrosis [32, 33]. Inhibition of CCR2 mitigated macrophage accumulation and renal atrophy in renovascular hypertension [34]. The intricate relationship between chronic kidney disease and hypertension seemed to have contributed to HF.

Thirdly, the phenotypic shift from differentiated to dedifferentiated vascular smooth muscle cells (VSMCs) contributes to the downregulation of contractile protein expression, heightened extracellular matrix production, and the induction of inflammatory cytokines. This transformation served as a major instigating factor in vascular remodeling during hypertension [62]. Vascular resident macrophages exhibit atheroprotective characteristics through the expression of CLEC4A2, facilitating the integration of monocyte-derived macrophages into the pool of vascular resident macrophages [30]. Moreover, extracellular vehicles (EVs) released by monocyte-derived macrophages have emerged as critical mediators in hypertension pathogenesis [63, 64]. These EVs transport a diverse cargo of microRNAs, proteins, and lipids that modulate cellular processes in VSMCs and endothelial cells, contributing to vascular remodeling and inflammation [63, 64]. EVs under hypertensive conditions have been shown to downregulate the phosphatidylinositol 3 phosphate kinase (PI3K)/protein kinase B (AKT)/mammalian target of rapamycin (mTOR) pathway in VSMCs, enhancing their invasive phenotype and promoting fibrosis [35, 36]. This intercellular communication mediated by EVs underscores their role in exacerbating vascular dysfunction in hypertension. On the other hand, in response to inflammation or microbial stimulation, TREM-2 has expressed on the surface of bone marrow–derived macrophages (BMDMs) and inhibits TLR-driven proinflammatory factor (such as TNF-β and IL-6) production and inflammatory responses [65]. Nevertheless, TREM-2^(high)^ macrophages aggravate atherogenesis via paracrine fibrotic signaling. TREM-2^(high)^ macrophages activated collagen secretion by fibroblasts, potentially mediated through paracrine phosphoprotein 1 (SPP1), also known as osteopontin [37, 38]. Therefore, the macrophages, particularly through the action of EVs and the TREM-2 signaling pathway, play crucial roles in the pathogenesis of hypertension and vascular remodeling.

Moreover, the interaction between macrophages and adipocytes within white adipose tissue (WAT) underscores the significant influence of adipose tissue macrophages on the development and progression of hypertension [66]. Specifically, macrophages in WAT secrete proinflammatory cytokines such as TNF-α and IL-6, which can induce local and systemic inflammation. This inflammatory milieu promotes insulin resistance in adipocytes, leading to an altered release of adipokines and free fatty acids [67]. These changes contribute to endothelial dysfunction, increased vascular resistance, and ultimately the elevation of blood pressure [68].

### 3.2. Hypertensive Cardiac Hypertrophy

**Figure 2:**
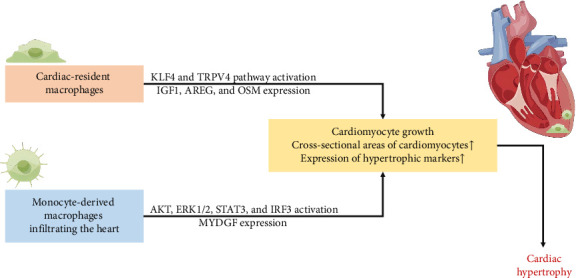
The role of macrophages in the development of hypertensive cardiac hypertrophy.

Prolonged pressure overload initiated adaptive cardiac hypertrophy, initially serving as a protective mechanism for the heart [69, 70]. However, in later stages, it transitioned into pathological cardiac hypertrophy, accompanied by cardiac dysfunction, ultimately fostering the development and progression of HF [71]. As illustrated in Table 1 and Figure 2, both resident macrophages and monocyte-derived macrophages in myocardial tissue played integral roles in the evolution of hypertensive heart hypertrophy [14, 39–48].

Myocardial adaptive responses, encompassing cardiomyocyte growth and increased cardiac mass, were imperative for withstanding hypertensive stress. On one hand, hypertension orchestrated a discerning in situ proliferation and transcriptional activation in specific cardiac resident macrophage states, guided by Kruppel-like factor 4 (KLF4) [40]. This directly associated with augmented cross-sectional areas of myocytes through a transient receptor potential vanilloid 4 (TRPV4)-dependent pathway, subsequently regulating the expression of growth factors such as insulin-like growth factor 1 (IGF1) and oncostatin M (OSM) [39–41]. The intricate interplay among the heart, brain, and kidneys underscores the indispensable homeostatic functions orchestrated by tissue macrophages and the SNS, essential for adaptive responses to cardiac stress. Kidney-secreted colony-stimulating factor 2 (CSF2) stimulated cardiac-resident macrophages, inducing the production of amphiregulin (AREG) [42]. Simultaneously, the deletion of AREG from cardiac-resident macrophages resulted in gap junction disorganization, leading to lethal arrhythmias during acute stresses such as right ventricle pressure overload and β-adrenergic receptor stimulation [14]. It became evident that cardiac resident macrophages played a critical role in maintaining cardiac impulse conduction by facilitating myocardial intercellular communication by gap junctions.

On the other hand, cardiac pressure overload recruited Ly-6C^(high)^ and Ly-6C^(low)^ monocyte-derived macrophages via CCR2 and CX3CR1, respectively; Ly-6C^(high)^ macrophages engaged in the digestion of damaged tissue, while Ly-6C^(low)^ macrophages promoted healing through myofibroblast accumulation, angiogenesis, and collagen deposition [25, 26]. CD8^(+)^ T-cells orchestrated the conversion of both cardiac resident macrophages and infiltrated macrophages into cardioprotective macrophages expressing growth factor genes such as AREG, OSM, and IGF1 [43]. Additionally, CCR2^(+)^ macrophages, expressing myeloid-derived growth factor (MYDGF), enhanced sarco/endoplasmic reticulum Ca^2+^-ATPase 2a (SERCA2a) expression in cardiomyocytes by boosting PIM1 expression and activity, playing a role in cardiac adaptation to persistent pressure overload [44].

However, the prolonged presence of Ly-6C^(high)^ macrophages in the heart led to pathological cardiac hypertrophy, relying on NF-κB transcription factors for expressing proinflammatory cytokines and upregulating multiple signals such as AKT, ERK1/2, STAT3, and calcium-regulated neurophosphatase A (CaNA) in cardiomyocytes [45, 46]. The uncontrolled influx of monocyte-derived macrophages correlated with increased expression of hypertrophic markers such as atrial natriuretic factor (ANF), brain natriuretic peptide (BNP), and β-myosin heavy chain (β-MHC). Myocardin-related transcription factor A (MRTF-A) potentially regulated macrophage trafficking, contributing to the pathogenesis of cardiac hypertrophy by activating integrin B2 (ITGB2) transcription [47]. Subsequently, these induced inflammatory responses and oxidative stress in cardiomyocytes. Therefore, the interruption of interferon regulatory factor 3 (IRF3)-dependent signaling could resulted in decreased inflammatory cell infiltration of the heart [48].

### 3.3. Hypertensive Cardiac Remodeling

**Figure 3:**
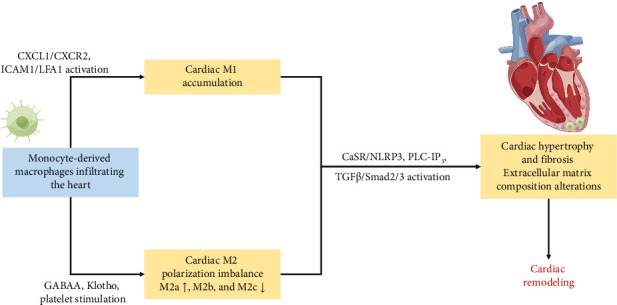
The role of macrophages in the development of hypertensive cardiac remodeling.

Cardiac hypertrophy induced ischemia and hypoxia in the heart tissue, subsequently triggering cardiac remodeling. The activation of cardiac macrophages was intricately linked to cardiac remodeling, as manifested by myocardial hypertrophy and alterations in extracellular matrix components [72]. As depicted in Table 1 and Figure 3, aberrant macrophage activation and immunological dysregulation were likely pivotal factors in cardiac remodeling [26, 45, 49–57].

Recruitment and adhesion of monocyte-derived macrophages to the vascular endothelium constituted critical steps in cardiac remodeling. Macrophage migration and activation were predominantly mediated by CXCL1-CXCR2 signaling [49]. In the previous study, blocking intercellular adhesion molecule-1 (ICAM-1) has been proved effective in preventing cardiac remodeling, by modulating adhesion and migration of lymphocyte function-associated antigen-1 (LFA-1) ^(+)^ monocyte-derived macrophages [45]. In addition, T-cell immune factors exert regulatory control over the recruitment of monocyte-derived macrophages, thereby exerting a profound influence on cardiac remodeling. Notably, the inhibition of CD40-TRAF6 signaling resulted in a notable decrease in the infiltration of macrophages and T-cells into the myocardium, concomitant with attenuated cardiac fibrosis and hypertrophy [50]. Monocyte-derived macrophages expedited the phenotypic transversion of cardiac fibroblasts, increasing collagen and extracellular matrix secretion through various molecular mechanisms, such as the calcium-sensing receptor (CaSR)/NLRP3 inflammasome via the phospholipase C-inositol phosphate 3 (PLC-IP3) pathway and the TGF-β/Smad2/3 pathway [51, 52].

The granulocyte-macrophage colony-stimulating factor (GM-CSF) induced the late stage of prolonged infiltration of monocyte-derived macrophages, promoting polarization from M1 towards M2. M2 further differentiated into M2a, M2b, and M2c based on stimuli and functions. Th2 cytokines (IL-4 or IL-13) activated M2a, secreting extracellular matrix components such as polyamines and collagen to facilitate extracellular matrix remodeling [73]. M2a matured into a CD206^(+)^ CD301^(+)^ M2a phenotype, eventually differentiating into fibroblasts [26, 53]. In contrast, M2b and M2c were involved in immunomodulation. Activation of the M2b subtype occurred through immune complexes, toll-like receptors (TLRs) agonists, or IL-1 receptor agonists [73]. Via the mitogen-activated protein kinase (MAPK) signaling pathway, M2b hindered cardiac fibroblasts' proliferation and migration, suppressing the expression of fibrosis-related proteins and preventing differentiation into myofibroblasts [74]. And M2c polarization, induced by IL-10, TGF-β, or glucocorticoids, regulated the extracellular matrix by downregulating the MAPK signaling pathway, expressing MMPs such as MMP-7, MMP-8, and MMP-9 and the tissue inhibitor of metalloproteinases 1 (TIMP1) [75, 76]. While M1 primarily polarized towards M2, the alterations in the collagen matrix composition of the myocardium during ventricular remodeling indicated that the profibrotic effect of M2a was not promptly regulated by M2b and M2c [54].

The recent researches have unveiled macrophage correlations with central brain neurotransmitters in cardiac remodeling. The central neurotransmitter gamma-aminobutyric acid subtype A (GABAA) was identified to increase Ly-6C^(high)^ macrophages infiltration in the myocardium and enhance the expression of MHC II molecules in Ly-6C^(low)^ macrophages [55]. Additionally, the study revealed that Ly-6C^(low)^ macrophages activated the AREG-induced AKT/mTOR signaling pathway. Ly-6C^(low)^ MHC II^(high)^ macrophages polarization resulted in elevated expression levels of osteopontin and TGF-β, contributing to myocardial hypertrophy and fibrosis [55]. Klotho, an antiaging protein, played a crucial role in alleviating ischemia-induced HF and kidney damage by inactivating NF-κB signaling and promoting macrophage M2 polarization [56]. Furthermore, platelets activated macrophages to upregulate MMP-7 expression via free molecules, leading to cardiac remodeling in uremic mice [57]. These emphasized the integral role of macrophages in the renal-derived cardiac remodeling mechanism.

## 4. Prevention Strategies

Exercise training emerged as a safer, more potent, and cost-effective therapeutic and preventive strategy for attenuating the progression from hypertension to HF. It induced multiple physiological adaptations in vascular and cardiomyocyte function through various intracellular mechanisms, activating extracellular and intracellular signaling pathways such as PI3K/AKT/mTOR, EGFR/JNK/SP-1, and nitric oxide (NO)-signaling [77–86]. Notably, exercise training also modulated macrophage function. Aerobic endurance exercise, for instance, heightened the release of inflammatory cytokines from M1, thereby enhancing the body's ability to combat infection [87, 88]. However, in individuals at greater cardiovascular risk, the ability of perivascular M1 to release inflammatory cytokines was reduced after reducing sedentary behavior and increasing walking time [89]. Consequently, this section explored the interplay between macrophage immune inflammatory activity and the positive physiological changes in vascular, cardiomyocyte, and skeletal muscle during exercise training (refer to Table 2).

First, aerobic endurance training emerged as a preventative measure against adverse vascular responses and the onset of hypertension, particularly in the context of chronic kidney disease. This was attributed, at least in part, to the inhibition of the infiltration and adhesion of monocyte-derived macrophages in the arterial vascular endothelium [90]. Intriguingly, when combined with β-blocker treatment, aerobic endurance training resulted in elevated macrophage levels in cardiac tissue, offering additional cardiovascular benefits compared to either intervention alone in hypertensive conditions [98]. These contrasting outcomes might be attributed to the possibility that the effects of exercise training on macrophage function were influenced by exercise-induced changes in the SNS [99].

Then, aerobic endurance training exerted a notable impact on the myocardial macrophage polarization, contributing to enhanced cardiac function. Specifically, aerobic walking exercise promoted the cardiac M2 phenotype, suppressing the immune response of Th1 cells through IL-10 secretion via the STAT3/S100A9 signaling pathway [91]. This augmentation served to improve cardiac function and impede the progression of HF. The amelioration in exercise tolerance induced by aerobic endurance training correlated with the mitigation of the inflammatory process involving monocyte-derived macrophages in dilated cardiomyopathy [92].

Finally, the M2 phenotype of skeletal muscle macrophages, facilitated by aerobic walking exercise, enhanced the expression of IGF-1 and other growth factors pivotal for muscle repair and regeneration, consequently improving exercise endurance [94–96]. Simultaneously, endurance training reduced M1 infiltration in skeletal muscle, thereby enhancing exercise tolerance. By inhibiting NF-κB signaling and activating the SIRT1/AMPKα/PGC1*α* axis, aerobic endurance training curtailed M1 accumulation in skeletal muscle, preventing atrophy of the tibialis anterior and gastrocnemius muscle groups [97]. Moreover, aerobic endurance training indirectly influenced macrophage function, leading to improved cardiac function. This form of training elevated circulating myonectin levels, indirectly suppressing the inflammatory response of macrophages through the S1P/cAMP/Akt-dependent signaling pathway [93]. This suppression served to alleviate apoptosis and inflammation in the heart.

## 5. Conclusion

This article provided a comprehensive examination of the pivotal role played by macrophage-related immune responses in the evolution from hypertension to HF across three distinct stages. Initially, macrophage-associated inflammatory responses in the brain and kidney were shown to elevate the risk of hypertension, renal atrophy, and vascular remodeling during the early phases of hypertension and its subsequent vasculopathy. Subsequently, cardiac resident and monocyte-derived macrophages exhibited distinct immunological responses as the cardiac load progressively intensifies, culminating in pathological myocardial enlargement during the second stage of hypertensive cardiac hypertrophy. Finally, in the third stage of hypertensive cardiac remodeling, aberrant immunological modulation of cardiac macrophages contributed to prolonged M1-mediated inflammatory loops and abbreviated M2-mediated anti-inflammatory repair loops. Furthermore, this study suggested that exercise training held promise in enhancing the prognosis for hypertensive heart disease patients by mitigating monocyte-derived macrophage tissue invasion and augmenting the polarization of protective macrophages.

In conclusion, while significant progress has been made in understanding these mechanisms, there are areas that warrant further investigation. Future research could explore the precise molecular pathways involved in macrophage polarization and their impact on hypertensive pathology. Additionally, understanding the role of macrophage-derived extracellular vesicles in hypertension and HF progression could unveil new therapeutic targets. Challenges remain in translating these findings into clinical practice, emphasizing the need for innovative strategies to modulate immune responses in cardiovascular disease effectively. Identifying these areas for further study will be crucial in advancing treatment options and improving patient outcomes.

## Figures and Tables

**Table 1:** Mechanism of the role of macrophages in the development and progression from hypertension to HF.

Macrophage type	Target location	Mechanism of action and function	Refs
*Stage 1. Hypertension and its subsequent vasculopathy*			
Resident macrophages	Brain vascular	1. Contributing to the development of hypertension via sympathetic activation	[28]
	Brain tissue	2. Involving in the development of hypertension via switching to the proinflammatory M1 state from a transient proliferative state	[29]
	Vascular tissue	3. Contributing to vascular health via CLEC4A2 activation	[30]
Monocyte-derived macrophages	Kidney vascular	4. Contributing to the development of hypertension via macrophage miR-106b-5p secretion from impaired vitamin D receptor signaling	[31]
	Kidney tissue	5. Involving in kidney injury and fibrosis via PI3Kγ, CXCR6, or CCR2 activation	[32–34]
	Vascular tissue	6. Involving collagen secretion via SPP1 signaling activation in TREM2^(high)^ macrophages 7. Contributing to the cell viability and invasion of VSMCs via PI3K/AKT/mTOR pathway inactivation	[35–38]

*Stage 2. Hypertensive cardiac hypertrophy*			
Resident macrophages	Myocardial tissue	1. Contributing to cardiomyocyte growth via KLF4 and TRPV4 pathway activation and the growth factor (IGF1, OSM, and AREG) expression	[14, 39–42]
Monocyte-derived macrophages	Myocardial tissue	2. Contributing to the development of pathological cardiac hypertrophy via NF-KB, AKT, ERK1/2, STAT3 and CaNA, ITGB2, and IRF3 pathway activation and MYDGF expression	[43–48]

*Stage 3. Hypertensive cardiac remodeling*			
Monocyte-derived macrophages	Myocardial tissue	1. Contributing to the development of cardiac remodeling via CXCL1-CXCR2 signaling, ICAM1-LFA1 signaling, the CaSR/NLRP3, the PLC-IP3 pathway, and TGFβ-Smad2/3 pathway activation 2. Involving in aberrant activation and immune dysregulation of macrophages via GM-CSF stimulation by M1 polarization M2 imbalance, as evidenced by increased M2a and decreased M2b and M2c	[26, 45, 49–57]

**Table 2:** Aerobic endurance training based on macrophages for preventing the development and progression of hypertension to HF.

Macrophage type	Target location	Mechanism of action and function	Refs
*Preventing adverse vascular responses and development of hypertension*			
Monocyte-derived macrophages	Vascular tissue	Reduction in the infiltration and adhesion of monocyte-derived macrophages in the arterial vascular endothelium	[90]

*Improving the heart structure and function to prevent HF progression*			
Monocyte-derived macrophages	Myocardial tissue	Polarization M1 into M2 and decreased peripheral markers of inflammation to improve the heart structure and function	[91–93]

*Reducing atrophy of the muscle groups to improve exercise endurance*			
Monocyte-derived macrophages	Skeletal muscle tissue	Polarization M1 into M2 and involving muscle repair and regeneration	[94–97]

## Data Availability

The data and materials used to support the findings of the study are available from the corresponding author upon request.
